# Non-insulinoma pancreatogenous hypoglycemia syndrome (NIPHS)/Nesidioblastosis as the underlying cause of recurrent hypoglycemia in a diabetic adult

**DOI:** 10.4322/acr.2023.451

**Published:** 2023-10-27

**Authors:** Samikshya Thapa, Kirandeep Kaur, Gajendra Kumar Yadav, Divya Kumari, Ravi Hari Phulware

**Affiliations:** 1 All India Institute of Medical Sciences AIIMS, Department of Pathology & Laboratory Medicine, Rishikesh, Uttarakhand, India; 2 All India Institute of Medical Sciences, Department of Endocrinology, Rishikesh, Uttarakhand, India

**Keywords:** Nesidioblastosis, Hyperinsulinism, Hypoglycemia, Pancreatic Diseases, Nutritional and Metabolic Diseases

## Abstract

Non-insulinoma pancreatogenous hypoglycemia syndrome (NIPHS), without previous bariatric surgery, is a rare form of hypoglycemia in adult patients and is associated with nesidioblastosis. Adult-onset nesidioblastosis in diabetic patients is rare and histologically identical to "non-insulinoma pancreatogenous hypoglycemia syndrome (NIPHS)". Nesidioblastosis is rare in adults and clinically and biochemically mimics Insulinoma. In the literature, there have only been four cases of adult nesidioblastosis that followed diabetes mellitus. We report a case of nesidioblastosis in a 36-year-old diabetic female presenting with dizziness, sweating, and palpitations for three years. Selective non-invasive techniques failed to detect a tumor. Based on the pursuit of an insulinoma, a distal pancreatectomy specimen was received at our laboratory, and a diagnosis of nesidioblastosis was made. She is currently on follow-up with a favorable outcome. The definitive diagnosis of nesidioblastosis is made on a histological basis. The preferred form of treatment is pancreatic surgical resection. Nesidioblastosis should be taken into consideration in cases where diabetes transforms into hyperinsulinemic hypoglycemia.

## INTRODUCTION

Nesidioblastosis is the infant’s primary cause of persistent hyperinsulinemic hypoglycemia (PHH). However, insulinoma is the major cause of persistent hyperinsulinemic hypoglycemia and rarely nesidioblastosis.^1^ It is characterized by pancreatic cell hyperplasia that impairs the regulation of insulin release from pancreatic beta-cells that are functionally deficient.^2^ This term was developed in 1971 by Yakovac et al.^2^ to describe pancreatic abnormalities in 12 newborns with intractable hypoglycemia. In 1975, the first adult patient with PHH not associated with an insulinoma was reported. Since then, a few hundred adult cases have been published. The majority of them were covered in specific case studies. Only four cases of nesidioblastosis in PHH with known diabetes mellitus have been described, making them even more uncommon.^3-5^

Due to its rarity and similarities to other pancreatic disorders, most notably insulinoma, which cannot be distinguished clinically and by imaging, establishing the diagnosis of nesidioblastosis in an adult is challenging.^3^ Herein, we present a case of nesidioblastosis in an adult in which the patient had a ten-year history of diabetes mellitus and had been experiencing recurrent hypoglycemia for the previous four years.

## CASE REPORT

An apparently healthy 36-year-old female presented with a history of several intermittent dizziness episodes, sweating and palpitations for three years. Initially, she experienced recurrent episodes every few days, but the frequency had increased to 3-4/ day over the last 6 months. These episodes happened most frequently in the early morning and improved after eating. She had a sudden-onset loss of consciousness twice and was hospitalized with hypoglycemia, which improved after dextrose administration. She was diagnosed with diabetes mellitus during pregnancy 10 years back, with glycosylated hemoglobin (HbA1c) of 8.2% (Reference range [RR]; prediabetic 5.7% to 6.4% and diabetes ≥6.5%) and hyperglycemia persisted post-delivery. She was taking metformin but had stopped 3 years back when the episodic symptoms started. Her symptoms continued despite stopping the medication and worsened progressively. She was also diagnosed with hypothyroidism and hypertension and is on regular medication.

The patient was admitted with an unremarkable systemic examination. Her vitals were within normal limits with BP 130/80 mm Hg. Routine biochemistry, including complete blood count and liver and renal function tests, were normal. Normal thyroid function tests and normal stimulated cortisol levels ruled out hypothyroidism and adrenal insufficiency. She underwent a supervised fast test in the hospital where during hypoglycemia (plasma glucose 42 mg/dl) (Reference range [RR]; 70-100mg/dl), serum insulin and C-peptide were 49.68 µIU/ml (RR; 5-25 µIU/ml) and 2.26 nmol/L (RR; 0.3-0.6 nmol/L) respectively; documenting endogenous hyperinsulinemia. Anatomical and functional scans were performed for the localization of the source of endogenous hyperinsulinemia.

**Figure 1:**
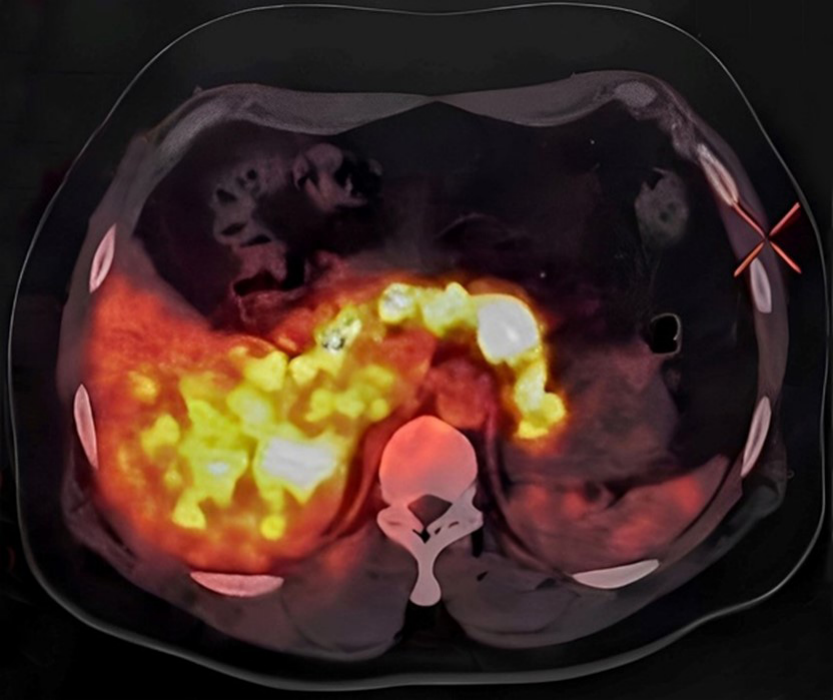
Ga 68 Exendin PET/CT: Showed diffuse uptake in the body and tail of pancreas.

Various imaging modalities like ultrasonography whole abdomen, contrast-enhanced computed tomography (CECT) abdomen, and Endoscopic ultrasound showed a normal pancreas with no suspicious lesion. A functional scan with Gallium 68 exendin PET/CT was performed, and it showed diffuse tracer uptake in the entire pancreas with few foci of increased tracer uptake (- 1.6 *2.8 cm, SUV max 13.4) in the distal body and tail of the pancreas (Figure 1). Diffuse uptake was suggestive of the hyperfunctioning pancreas as seen in nesidioblastosis, but few loci of increased uptake at the distal end couldn’t rule out an insulinoma.

The patient was operated on. The intraoperative inventory found no suspicious pancreatic lesions; however, a distal pancreatectomy was performed.

**Figure 2:**
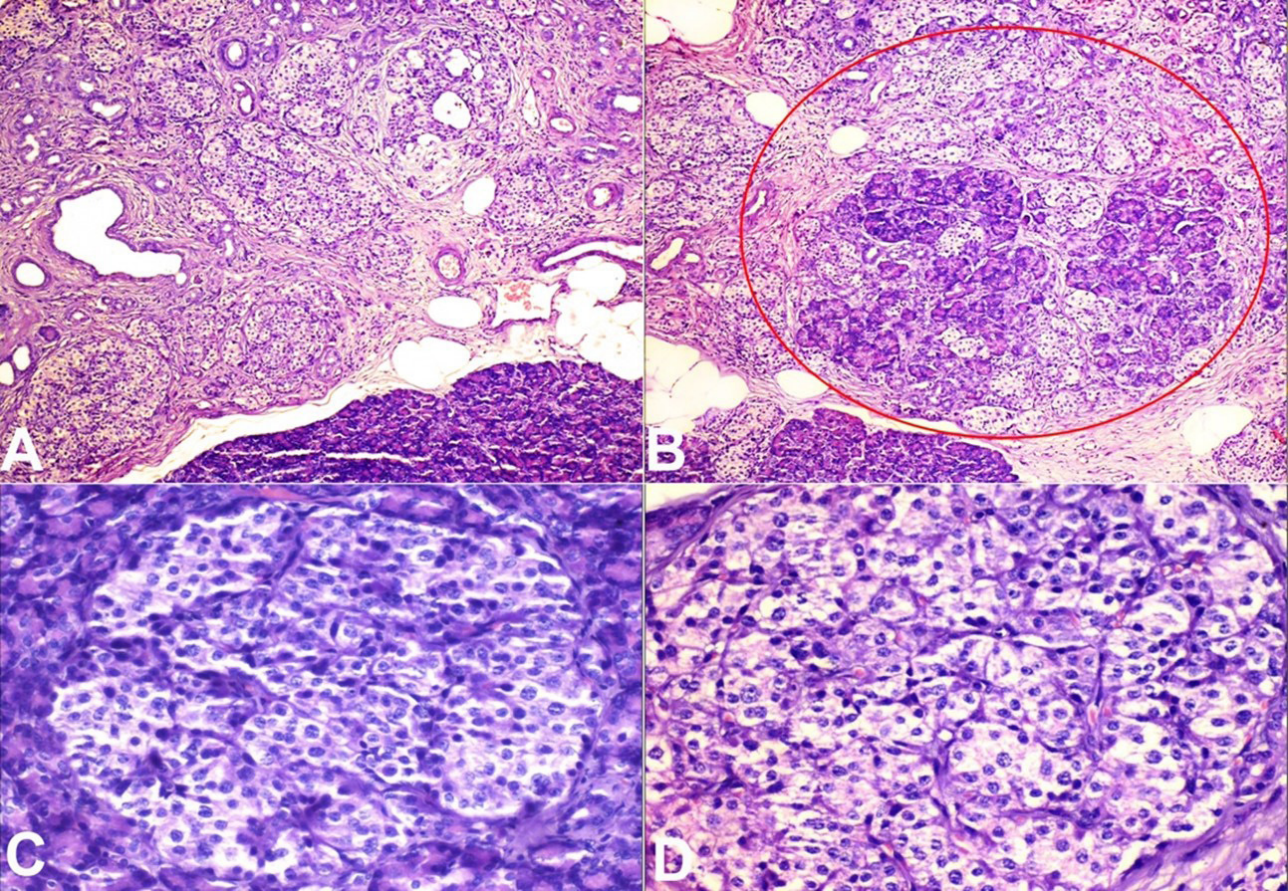
Photomicrographs of the pancreas. **A** and **B -** Diffuse proliferation of variably sized islet cells arranged in lobules and forming tubule-insular complex (red circle magnification); **C** and **D -** Enlarged Islet cells showing irregular nuclear contour, mildly enlarged nuclei with clear cytoplasm (H&E x400).

A frozen specimen of distal pancreatectomy was received at our laboratory and was proceeded for analysis. The specimen measured 7x5x1 cm, weighing 15 g. The outer surface and the parenchyma on the serial section were unremarkable. No definitive growth was identified. Microscopic examinations showed maintained lobular exocrine pancreatic parenchyma architecture, with a proliferation of islet cells arranged in variably sized with lobulated patterns (Figures 2A and 2B). Individual cells were mildly irregular in shape with mildly increased size, centrally placed nuclei with a moderate amount of clear cytoplasm (Figures 2C and 2D).

**Figure 3:**
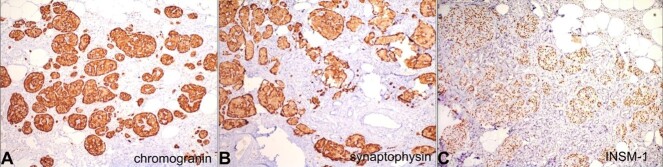
Photomicrographs of the pancreas. Positive immunohistochemical reactions for **A**  **-** Chromogranin; **B -** Synaptophysin, **C -** ISNM-1.

No mitotic figures and necrosis were identified. On immunohistochemistry, the enlarged and irregular islet cells were immune-reactive with chromogranin, synaptophysin, and Insulinoma-associated protein 1 (INSM1) (Figures 3A, 3B, and 3C). Based on histomorphological features combined with clinical and radiological findings, the final diagnosis was released as diffuse nesidioblastosis.

The post-operative period was uneventful, and the patient is on follow-up with a favorable outcome. She only had an episode of hypoglycemia in three months after surgery and is being managed with oral Octreotide and Verapamil.

## DISCUSSION

Nesidioblastosis is a rare illness that typically causes post-prandial hypoglycemia and endogenous hyperinsulinism. George Laidlaw^1,3^ chose the Greek word for islet, nesidion, and combined it with the word "blastos" to underline that in this disease, the cells differentiate and bud from ducts to produce new islet cells of Langerhans in the exocrine pancreatic ductal epithelium. This condition is known as nesidioblastosis.^6^ It is also known by the term islet cell hyperplasia, ductuloinsular proliferation, islet cell adenomatosis, and nesidioplasia. Adults can experience it in either sex between 28 and 63 years old, with 48 as the median. Most frequently, it occurs in childhood ~ 40%, unlike adults, where it accounts for only 0.5-5% of cases of organic hyperinsulinism.^7^

Approximately 95% of nesidioblastosis cases are sporadic; nevertheless, associations with numerous pancreatic islet cell adenomas, Beckwith-Weidemann syndrome, adults undergoing bariatric surgery, and Zollinger-Ellison syndrome have been noted. It is plausible that diffuse nesidioblastosis in adults may be caused by the same or comparable gene abnormalities as diffuse nesidioblastosis in infancy because the morphologic characteristics of nesidioblastosis in adults are similar to those in neonates.^8^

According to our understanding of the etiology, various genetic mutations are responsible for the hyperplasia of pancreatic beta cells. The two genes encoding the ABCC8 (formerly known as SUR1 sulfonylurea receptor1) and KCNJ11 (formerly Kir6.2) proteins, which generate the ATP-sensitive potassium channel in the cell membrane of the beta cell, are the most significant genetic defects causing the diffuse form of nesidioblastosis. Mutations in these genes are located on chromosome 11p14-15.1, causing alteration in the channel, favoring its inactivity. Eventually, the closure results in calcium entry and cell membrane depolarization, which enable continuous secretion of insulin. The majority of cases exhibit an autosome and recessive inheritance.^1,9^ Rarely, gain-of-function mutations of the glucokinase (GCK) and glutamate dehydrogenase (GLUD1) genes are detected, which are of autosomal dominant transmission. The focal form of the disease is characterized by a random loss of maternally imprinted growth inhibitory genes at chromosome 11p15 and a paternally inherited inactivating mutation of the SUR1 or Kir6.2 gene.^10,11^

It has been strongly discussed whether the fasten test should be use to diagnose nesidioblastosis. The diagnosis has been based on the following criteria.^10^

1. Insulinoma exclusion using all available clinical diagnostic tools.
2. A review of the pancreatic tissue specimens' pathology
3. A combination of various histopathological criteria (Table 1) marked as major and minor criteria, has recently been published to establish the diagnosis of a diffuse adult nesidioblastosis

**Table 1:** Histopathological Criteria for the Diagnosis of Diffuse Nesidioblastosis in Adults^9,12^

**MAJOR CRITERIA**	**MINOR CRITERIA**
-Exclusion of an insulinoma by macroscopic, microscopic, and immunohistochemical examination	-Irregular shape and occasional enlargement of islets
-Multiple b-cells with an enlarged and hyperchromatic nucleus and abundant clear cytoplasm	-Increased number of islets
-Islets with normal spatial distribution of the various cell types	-Lobulated islet structure
-No proliferative activity of endocrine cells	-Macronucleoli in b-cells

Nesidioblastosis cases have primarily been reported in the young (around 40%), with only a few occurrences in adults. In 2001, Witteles et al.^11^ described five cases of adult-onset nesidioblastosis that showed diffuse islet tissue growth, resulting in islets of various sizes.^11,13^ Similarly, a study by Kaczirek and Niederle^13^ diagnosed adult nesidioblastosis with five individuals who met all the histology criteria, similarly to our study. A survey conducted by Raffel et al.^14^ found four out of one hundred twenty cases of hyperinsulinemic hypoglycemia classified as adult nesidioblastosis, with just one case having concurrent diabetes.

Histopathological and clinical characteristics of nesidioblastosis in adults may be challenging due to its rarity and the lack of standardized diagnostic criteria. However, some features commonly associated with nesidioblastosis in adults are mentioned above (Table 1). In the present case, all four major criteria were fulfilled along with minor criteria, and the definitive diagnosis was made. Patients with adult nesidioblastosis appear to have grossly normal-looking pancreatic tissue.^6^ Upon histopathologic study, the lobular architecture of the exocrine parenchyma is typically intact, and the modification in the endocrine pancreas may vary from patient to patient. Nesidioblastosis is characterized histologically by abnormal beta-cell proliferation throughout the pancreas. These abnormal beta cells, also known as nesidioblastosis, cause excessive insulin secretion, resulting in hyperinsulinism and, eventually, hypoglycemia.^3-5^ Nesidioblastosis can be focal or diffuse. The focal type is characterized by nodular hyperplasia of islet-like cell clusters, including ductuloinsular complexes, and hypertrophy of beta cells with giant nuclei in specific pancreatic regions. The diffuse form refers to the involvement of the whole pancreatic gland with irregularly sized islets and ductuloinsular complexes, containing hypertrophied beta-cells.^15,16^ Histopathological examination is a useful feature that can assist in diagnosing coupled with Immunohistochemistry.

Adult nesidioblastosis diagnosis and therapy are still up for debate. There are no imaging methods to distinguish focal and diffuse organic hyperinsulinism. Due to the usual small size (less than 10 mm), the insulin-producing tumors may go undetected before surgery.^17^ Surgical resection is the mainstay of treatment for adult nesidioblastosis. Since nesidioblastosis has recently been demonstrated to occur in up to 5% of PHH patients, it must be taken into account in all instances without localized insulinoma. In a recurrence, more pancreatic resection via secondary surgery is required.^18^ Due to the patient’s young age, in this case, a total pancreatectomy was not performed.

## CONCLUSION

The current case presents a rare and unusual combination of adult nesidioblastosis along with diabetes. To date, there have been only four documented cases of this unique co-occurrence of organic hyperinsulinism and diabetes (Table 2).

**Table 2:** Reported cases of nesidioblastosis with persistent hyperinsulinemic hypoglycemia (PHH) in known diabetes mellitus patients

Reference	PMID	Age	Gender	History of Diabetes Mellitus
^3^	11126710	57	Female	4 months
^4^	19829681	84	Male	6 years
^6^	8721942	50	Male	5 years
^14^	17263973	40	Male	9 months

Nesidioblastosis is a rare but clinically significant cause of hypoglycemia in the adult population, and it should always be taken into account in patients who have been preoperatively presumed to have an insulinoma. The localization of pathologic elevated insulin secretion would be a diagnostic tool for the surgeon to prevent blind pancreatic resection due to the futility of contemporary imaging modalities in these patients. However, only surgical pathology can confirm this diagnosis, which is still one of exclusion.
